# Efficacy and safety comparing prasugrel/ticagrelor and clopidogrel in Hong Kong post‐acute coronary syndrome patients–A 10‐year cohort study

**DOI:** 10.1002/clc.23653

**Published:** 2021-05-26

**Authors:** Amy S. M. Lam, Bryan P. Y. Yan, Vivian W. Y. Lee

**Affiliations:** ^1^ Department of Medicine & Therapeutics, Faculty of Medicine The Chinese University of Hong Kong Shatin Hong Kong; ^2^ Centre for Learning Enhancement And Research (CLEAR) The Chinese University of Hong Kong Shatin Hong Kong

**Keywords:** acute coronary syndrome, dual antiplatelet therapy, efficacy, P2Y12 inhibitor, safety

## Abstract

**Background:**

Clinical evidence of prasugrel/ticagrelor in dual antiplatelet therapy (DAPT) in Asian acute coronary syndrome (ACS) population remains inconclusive. We aimed to compare the clinical efficacy and safety of prasugrel/ticagrelor compared to clopidogrel as part of DAPT in Hong Kong ACS population for 10 years.

**Hypothesis:**

Prasugrel/ticagrelor, compared to clopidogrel, reduces risk of major adverse cardiovascular event (MACE) in Hong Kong ACS population.

**Methods:**

The retrospective observational cohort study included patients admitted to seven institutions under Hospital Authority Hong Kong with diagnosis of ACS during 2008–2017. Risk of MACE, defined as composite of cardiovascular (CV) death, non‐fatal myocardial infarction (MI) and non‐fatal stroke, and risk of any bleeding leading to hospitalization were examined. Baseline characteristics difference was adjusted by propensity score (PS) matching. Adjusted Cox regression model was used to estimate hazard ratio of interested outcome.

**Results:**

In PS matched cohort including 944 patients in each group, MACE risk reduction of 40% from 1 year to 5 years after index ACS event was observed in prasugrel/ticagrelor group (HR 0.60, 95% CI 0.39–0.91, p = .015). The risk reduction was highly driven by MI reduction (HR 0.54, 95% CI 0.33–0.91, p = .019). Lower bleeding risk was observed in prasugrel/ticagrelor group compared to clopidogrel from 1 year to 5 years (HR 0.46, 95% CI 0.21–1.00, p = .051).

**Conclusions:**

Prasugrel/ticagrelor showed MACE risk reduction over clopidogrel as part of DAPT up to 5 years after index event, while prasugrel/ticagrelor was not associated with increased bleeding risk.

## 1 INTRODUCTION

Dual antiplatelet therapy (DAPT) is indicated in post‐acute coronary syndrome (ACS) patients, while use of prasugrel or ticagrelor is recommended over clopidogrel as suggested by international guideline recommendation.^1^, ^2^ Major clinical trials depicting benefit of prasugrel or ticagrelor over clopidogrel were mainly based on Caucasian population.^3^ The landmark study for prasugrel in ACS patients, TRITON‐TIMI 38, did not include Asian population, while in another prasugrel study for NSTE‐ACS patients, TRILOGY‐ACS, there was 8.1% (*n* = 571) of subjects from East Asia.^4^, ^5^ The PLATO study for ticagrelor recruited 1096 Asian patients to the study, which composed 5.9% of total cohort.^6^ Yet, Asian population has lower thrombogenicity compared to Caucasian population.^3^ Current evidence from real‐world data and meta‐analysis remained inconclusive on use of prasugrel or ticagrelor over clopidogrel in Asian population.^7^, ^8^, ^9^, ^10^ There has not been a comprehensive review on clinical outcome of DAPT with prasugrel or ticagrelor over DAPT with clopidogrel in Hong Kong ACS population. This study aimed to compare the clinical efficacy and safety of prasugrel or ticagrelor over clopidogrel as part of DAPT in Hong Kong post‐ACS patients for 10 years.

## 2 METHODOLOGY

The retrospective observational cohort study included patients, aged 18 years old or above, admitted to institutions under New Territories East cluster (NTEC), Hospital Authority (HA), Hong Kong with diagnosis of ACS from 01 January 2008 to 31 December 2017 and discharged with DAPT with clopidogrel, prasugrel or ticagrelor. Medical information of recruited subjects, including demographic, clinical and procedural and dispensing record, were retrieved through HA clinical data analysis and report system (CDARS) until 31 December 2018. Admission diagnosis, baseline demographics and comorbidities, and procedures were identified based on International Classification of Disease, 9th Revision Clinical Modification (ICD‐9‐CM) codes. List of ICD‐9‐CM codes used was attached in Table S1. Focus of current study was on post‐discharge long term effect of antiplatelet therapy management. Patients who died at index hospitalization or missing drug dispensing record were excluded. Drug exposure during index hospitalization was captured only if the drug continued upon hospitalization discharge. Medication use only during index hospitalization yet not continued upon discharge was not described in this study. Time to event was defined as time from index hospitalization admission to interested event date, with censorship applied to patient's death or study end on 31 December 2018. The study design was based on intention to treat (ITT) approached. Dispensing duration of a drug was defined as sum of dispensing episode within study period after patient's index admission before interested event outcome or censorship. Discontinuation of a dispensing episode was defined as dispensing gap of a drug more than 28 days. As dispensing history was used in the study, to avoid any mistaken record of switching antiplatelet treatment (especially P2Y12 receptor antagonist) during an early follow‐up, duration of continued drug exposure instead of number of daily doses dispensed was used.

Recruited subjects were categorized into DAPT with clopidogrel or DAPT with prasugrel or ticagrelor group. The primary efficacy outcome of the study was major adverse cardiovascular event (MACE), which was defined as composite of cardiovascular death, non‐fatal myocardial infarction (MI, including non‐ST‐elevation MI (NSTEMI) and ST‐elevation MI (STEMI)), and non‐fatal ischemic stroke. The secondary efficacy outcome was occurrence of each primary efficacy outcome and all‐cause mortality. The primary safety outcome was defined as occurrence of any bleeding leading to hospitalization, including gastrointestinal bleeding, cerebral bleeding, bleeding of joint, pericardial bleeding, hematuria, abnormal bleeding of female genital tract, hemoptysis, epistaxis and rupture of blood vessels.

Descriptive statistics were presented as counts (percentage) for qualitative variable and mean (*SD*) for quantitative variable unless otherwise specified. Comparison on qualitative variables was made between groups with Pearson's chi square test or Fisher's exact test if appropriate. Difference on quantitative variables between groups was compared with Student's t‐test. To address baseline difference of two groups, propensity score matching was used. Propensity score was calculated by logistic regression model with variables including sex, age, comorbidities of hypertension, diabetes, heart failure, chronic kidney disease, liver disease, arrhythmia, dyslipidemia, history of stroke, ischemic heart disease, history of ACS episode, anemia, history of bleeding event, status of percutaneous coronary intervention (PCI) or coronary artery bypass graft (CABG) during index hospitalization, use of fibrinolytics or glycoprotein (GP) IIb/IIIa receptor inhibitors during index hospitalization, dispensing of oral anticoagulant (OAC, including warfarin, dabigatran etexilate, rivaroxaban, apixaban and edoxaban), histamine‐2 receptor antagonist (H2RA, including famotidine and ranitidine) or proton pump inhibitor (PPI, including pantoprazole, rabeprazole, esomeprazole, lansoprazole and omeprazole) during index hospitalization discharge. Matching algorithm of 1‐to‐1 nearest neighbor method was adopted. Kaplan–Meier estimator was used to calculated time to event. Cox proportional hazard model was used to estimate hazard ratio of outcome using prasugrel or ticagrelor over clopidogrel, with adjustment of duration of DAPT and duration of concurrent use of OAC, H2RA or PPI. To check the proportional hazard assumption of models, Schoenfeld residuals were inspected to ensure insignificance between residuals and time. All statistical analyses were performed using R (version 3.5.3).

Subgroup analyses on (1) 1st time ACS patients, (2) post‐PCI patients and (3) patients aged 65 or above was done for sensitivity test. Further sensitivity analysis was also done with per protocol (PP) approach, of which censorship applied when there was switching of P2Y12 receptor antagonist during the follow up period after index hospitalization discharge. Sensitivity analysis was done with propensity score weighting, with or without trimming, to verify the robustness of results. Ethics approval was obtained from the Joint Chinese University of Hong Kong–NTEC Clinical Research Ethics Committee (CREC no.: 2019.090).

## 3 RESULTS

### 3.1 Patients characteristics

**FIGURE 1:**
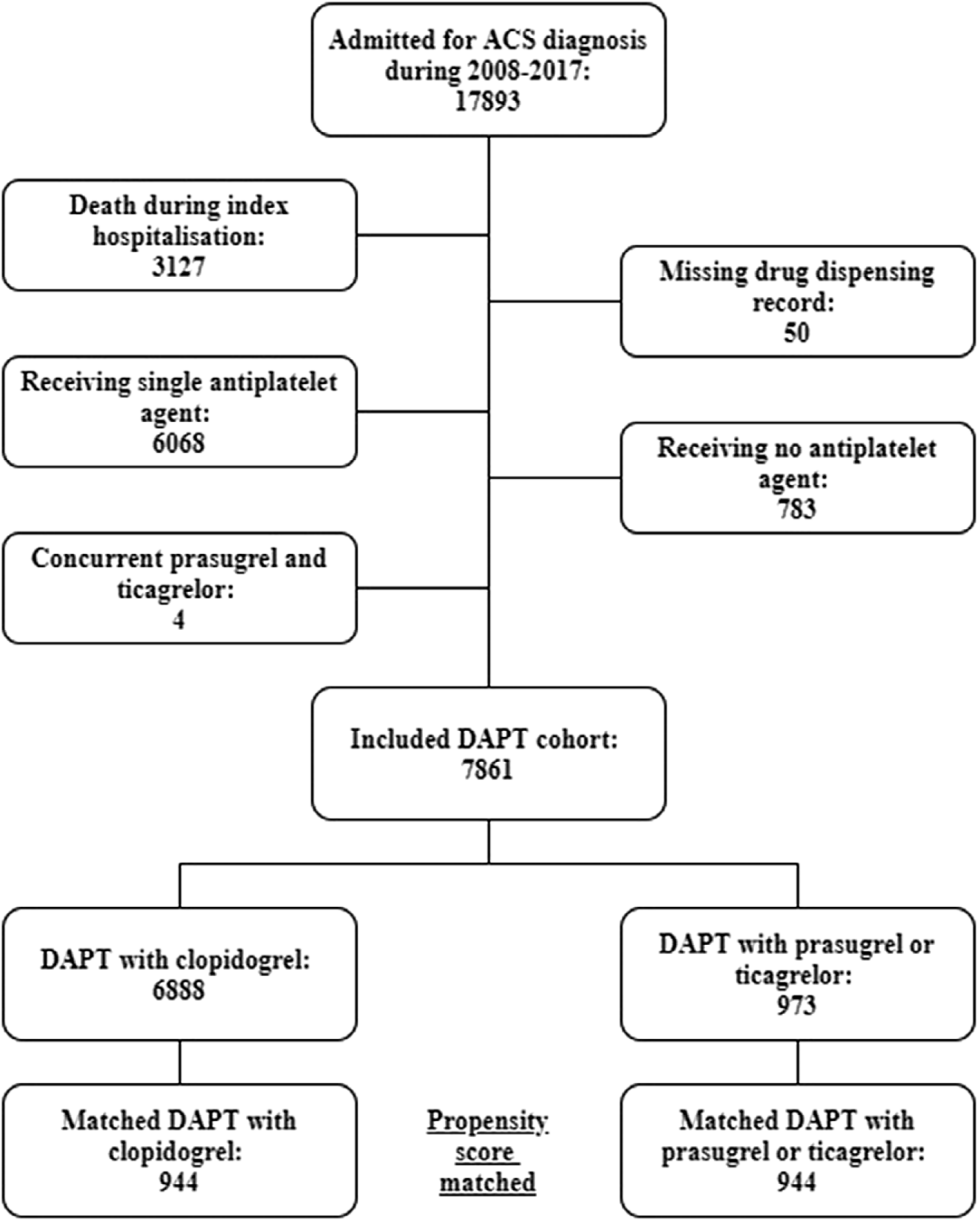
Study cohort diagram on data selection for the analysis

Of those 17 893 patients admitted for ACS diagnosis during 1st January 2008 to 31st December 2017, 7861 patients received DAPT. Within the included DAPT cohort, 6888 (87.6%) of subjects received clopidogrel, while 973 (12.4%) received prasugrel or ticagrelor. The complete cohort selection was shown in Figure 1.

The matched cohort consisted 944 subjects receiving clopidogrel and 944 subjects receiving prasugrel or ticagrelor. The baseline characteristics of unmatched and matched cohort were described in Table 1. No statistically significant difference in patients' demographics and comorbidities was observed after propensity score matching.

**TABLE 1:** Baseline characteristics of clopidogrel and prasugrel/ticagrelor groups

	Pre‐matching				Post‐matching			
	Clopidogrel *N* = 6888	Prasugrel/ticagrelor *N* = 973	p value	Standardized difference (%)	Clopidogrel *N* = 944	Prasugrel/ticagrelor *N* = 944	p value	Standardized difference (%)
Male	5154 (74.8)	831 (85.4)	<.001	26.7	795 (84.2)	802 (85.0)	0.702	2.1
Age, mean (*SD*)	65.3 (12.6)	60.1 (9.8)	<.001	45.9	60.1 (12.0)	60.1 (9.9)	0.948	0.3
Index ACS: STEMI	2156 (31.3)	530 (54.5)	<.001	48.1	471 (49.9)	501 (53.1)	0.182	6.4
Baseline comorbidities								
HTN	1345 (19.5)	102 (10.5)	<.001	25.5	93 (9.9)	102 (10.8)	0.545	3.1
DM	1093 (15.9)	80 (8.2)	<.001	23.6	90 (9.5)	80 (8.5)	0.469	3.7
HF	322 (4.7)	14 (1.4)	<.001	18.9	17 (1.8)	14 (1.5)	0.717	2.5
CKD	253 (3.7)	10 (1.0)	<.001	17.5	16 (1.7)	10 (1.1)	0.323	5.5
Liver Disease	61 (0.9)	3 (0.3)	.092	7.5	2 (0.2)	3 (0.3)	1	2.1
Arrhythmia	134 (2.0)	10 (1.0)	.061	7.6	10 (1.1)	10 (1.1)	1	0.0
Dyslipidemia	586 (8.5)	49 (5.0)	<.001	13.8	44 (4.7)	49 (5.2)	0.671	2.4
Ischemic stroke	194 (2.8)	16 (1.6)	.044	7.9	17 (1.8)	16 (1.7)	1	0.8
Hemorrhagic stroke	22 (0.3)	2 (0.2)	0.76	2.2	2 (0.2)	2 (0.2)	1	0.0
IHD	1004 (14.6)	72 (7.4)	<.001	23.1	73 (7.7)	72 (7.6)	1	0.4
Prior episode of ACS	433 (6.3)	32 (3.3)	<.001	14.1	31 (3.3)	32 (3.4)	1	0.6
Anemia	284 (4.1)	15 (1.5)	<.001	15.6	15 (1.6)	15 (1.6)	1	0.0
History of bleeding	489 (7.1)	41 (4.2)	.001	12.5	38 (4.0)	40 (4.2)	0.908	1.1
During index hospitalization								
GP IIb/IIIa	469 (6.8)	262 (26.9)	<.001	55.8	233 (24.7)	234 (24.8)	1	0.2
Fibrinolytics	166 (2.4)	23 (2.4)	1	0.3	19 (2.0)	23 (2.4)	0.64	2.9
PCI	2586 (37.5)	695 (71.4)	<.001	72.3	640 (67.8)	666 (70.6)	0.213	6.0
CABG	188 (2.7)	72 (7.4)	<.001	21.4	77 (8.2)	71 (7.5)	0.669	2.4
Concurrent medication during index hospitalization discharge								
H2RA	2776 (40.3)	308 (31.7)	<.001	18.1	307 (32.5)	307 (32.5)	1	0.0
PPI	2643 (38.4)	573 (58.9)	<.001	41.9	541 (57.3)	544 (57.6)	0.926	0.6
OAC	150 (2.2)	5 (0.5)	.001	14.5	7 (0.7)	5 (0.5)	0.772	2.7

Abbreviations: ACS, acute coronary syndrome. CABG, coronary artery bypass grafting. CKD, chronic kidney disease. DM, diabetes. GP IIb/IIIa, glycoprotein IIb/IIIa receptor inhibitor. HF, heart failure. HTN, hypertension.H2RA, histamine‐2 receptor antagonist. IHD, ischemic heart disease. OAC, oral anticoagulant. PCI, percutaneous coronary intervention. PPI, proton pump inhibitor. *SD*, standard deviation STEMI, ST‐elevation myocardial infarction.

*Note*: Data are shown as frequency (percentage) unless specified.

The duration of DAPT treatment was shorter in clopidogrel group (clopidogrel 308 days vs prasugrel/ticagrelor 366 days, p < .001), using MACE as outcome event. Duration of subjects follow up was longer in clopidogrel group (clopidogrel 1656 days vs. prasugrel/ticagrelor 1014 days, p < .001). Duration of DAPT and concurrent use of other medication were different in clopidogrel and prasugrel/ticagrelor group, and thus Cox regression model was adjusted accordingly with these variables (Table S2).

**FIGURE 2:**
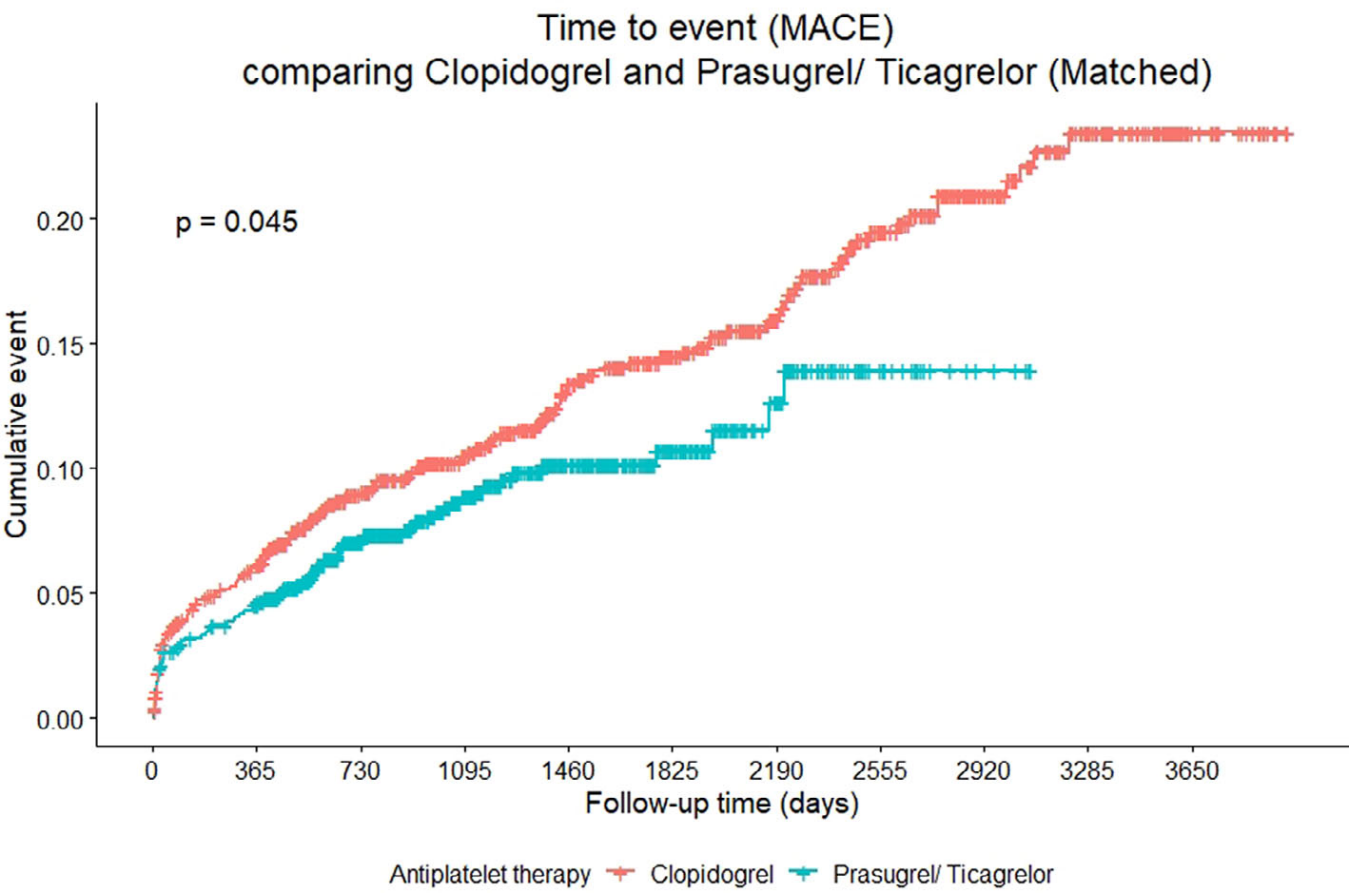
Kaplan–Meier curve for major adverse cardiovascular event (MACE) in matched cohort

After propensity score matching, the event rate of MACE was 6.0% (95% CI 4.5–7.5%) in clopidogrel group and 4.6% (95% CI 3.2–5.9%) in prasugrel/ticagrelor group at 1 year, 14.5% (95% CI 11.9–16.9%) in clopidogrel group and 10.7% (95% CI 8.2–13.2%) in prasugrel/ticagrelor group at 5 year (Table 2 and Figure 2). The MACE was highly driven by MI. The event rate of all‐cause mortality was 4.4% (95% CI 3.1–5.8%) in clopidogrel group and 2.0% (95% CI 1.1–2.9%) in prasugrel/ticagrelor group at 1 year, 13.6% (95% CI 11.2–16.0%) in clopidogrel group and 5.8% (95% CI 4.0–7.5%) in prasugrel/ticagrelor at 5 year. After adjustment with duration of concurrent medication and DAPT duration, 40% risk reduction in MACE from 1 year to 5 years was observed in prasugrel/ticagrelor group (HR 0.60, 95% CI 0.39–0.91, p = .015). The risk reduction during the period was highly driven by MI reduction (HR 0.54, 95% CI 0.33–0.91, p = .019). Overall risk reduction in CV death (HR 0.47, 95% CI 0.25–0.91, p = .024) and ischemic stroke (HR 0.49, 95% CI 0.24–0.99, p = .048) were also observed in prasugrel/ticagrelor group. Prasugrel/ticagrelor reduced overall risk of all‐cause mortality (HR 0.49, 95% CI 0.35–0.70, p < .001), particularly from 1 year to 5 years (HR 0.42, 95% CI 0.26–0.68, p < .001). Table 3 showed results from Cox regression models with various event outcomes. All p‐values for Schoenfeld residual test after period stratification of during the first year, from 1 year to 5 years, and after 5 years were insignificant (p > .05), indicating the valid assumption of proportional hazard.

**TABLE 2:** Event rate by Kaplan–Meier analysis comparing clopidogrel and prasugrel/ticagrelor group in matched cohort using intention to treat approach

		Clopidogrel (*N* = 944)			Prasugrel/Ticagrelor (*N* = 944)		
	Time after index ACS event	Number at risk	Number of events (cumulative)	Event rate (%)	Number at risk	Number of events (cumulative)	Event rate (%)
MACE	1 year	860	56	6.0 (4.5–7.5)	889	43	4.6 (3.2–5.9)
	5 years	434	117	14.5 (11.9–16.9)	140	79	10.7 (8.2–13.2)
	10 years	26	145	23.4 (19.2–27.5)	Nil	Nil	Nil
CV Death	1 year	902	11	1.2 (0.5–1.9)	925	6	0.6 (0.1–1.2)
	5 years	488	31	4.2 (2.7–5.6)	155	14	1.9 (0.9–2.9)
	10 years	35	35	5.2 (3.4–6.9)	Nil	Nil	Nil
MI	1 year	865	43	4.6 (3.3–6.0)	893	33	3.5 (2.3–4.7)
	5 years	442	87	10.7 (8.5–12.8)	143	57	7.5 (5.5–9.4)
	10 years	28	109	18.5 (14.4–22.3)	Nil	Nil	Nil
Ischemic stroke	1 year	893	11	1.2 (0.5–1.9)	921	4	0.4 (0.0–0.8)
	5 years	476	23	2.9 (1.7–4.1)	152	11	1.8 (0.4–3.2)
	10 years	32	29	4.8 (2.8–6.8)	Nil	Nil	Nil
All‐cause mortality	1 year	902	42	4.4 (3.1–5.8)	925	19	2.0 (1.1–2.9)
	5 years	488	109	13.6 (11.2–16.0)	155	43	5.8 (4.0–7.5)
	10 years	35	136	21.4 (17.4–25.1)	Nil	Nil	Nil
Bleeding	1 year	880	28	3.0 (1.9–4.1)	905	23	2.5 (1.5–3.4)
	5 years	466	54	6.8 (5.0–8.6)	147	32	4.5 (2.7–6.2)
	10 years	30	64	10.3 (7.2–13.2)	Nil	Nil	Nil

Abbreviations: ACS, acute coronary syndrome. CV, cardiovascular. MACE, major adverse cardiovascular event. MI, myocardial infarction.

*Note*: Event rate was presented with 95% confidence interval.

**TABLE 3:** Cox regression comparing clopidogrel versus prasugrel/ticagrelor in matched cohort

Outcome			Time after ACS event					
	Overall		<1 year		1–5 years		>5 years	
	HR (95% CI)	p value	HR (95% CI)	p value	HR (95% CI)	p value	HR (95% CI)	p value
MACE	0.77 (0.57–1.02)	.071	1.25 (0.83–1.87)	0.283	**0.60 (0.39–0.91)**	**.015**	0.71 (0.21–2.38)	0.577
CV Death	**0.47 (0.25–0.91)**	**.024**	0.72 (0.26–2.02)	0.536	0.45 (0.19–1.03)	.058	N/A	N/A
MI	0.77 (0.55–1.08)	0.135	1.26 (0.79–2.00)	0.325	**0.54 (0.33–0.91)**	**.019**	0.992 (0.286–3.43)	0.989
Ischemic stroke	**0.49 (0.24–0.99)**	**.048**	0.53 (0.16–1.72)	0.290	0.62 (0.24–1.61)	0.323	1.02 (0.12–8.78)	0.988
All‐cause mortality	**0.49 (0.35–0.70)**	**<.001**	0.64 (0.37–1.11)	0.114	**0.42 (0.26–0.68)**	**<.001**	1.00 (0.34–2.96)	1.000
Bleeding	0.72 (0.46–1.13)	0.155	1.07 (0.60–1.90)	0.815	0.46 (0.21–1.00)	.051	N/A	N/A

Abbreviations: ACS, acute coronary syndrome. CV, cardiovascular. MACE, major adverse cardiovascular event. MI, myocardial infarction.

*Note*: Clopidogrel group was used as reference. Value in **bold** were with statistical significance (p < .05). Variables for adjusted model included duration of DAPT, duration of H2RA, duration of PPI and duration of OAC.

After propensity score matching, bleeding rate was 3.0% (95% CI 1.9–4.1%) in clopidogrel group and 2.5% (95% CI 1.5–3.4%) in prasugrel/ticagrelor group at 1 year, 6.8% (95% CI 5.0–8.6%) in clopidogrel group and 4.5% (95% CI 2.7%–6.2%) in prasugrel/ticagrelor group at 5 years. After adjustment, lower bleeding risk was observed from 1 year to 5 years in prasugrel/ticagrelor group (HR 0.46, 95% CI 0.21–1.00, p = .051).

Subgroup analyses in the first time ACS patients and post‐PCI patients showed prasugrel/ticagrelor reduced MACE risk and reduced MI risk from 1 year to 5 years compared to clopidogrel, similar to primary outcome analysis (Table S3). In patients aged 65 or above, reduction of MACE and MI using prasugrel/ticagrelor were not observed, while overall CV death and overall ischemic stroke risk were reduced. In all three subgroup analyses, reduction in all‐cause mortality was observed, both overall and from 1 year to 5 years. This indicated the robustness of all‐cause mortality reduction in prasugrel/ticagrelor group compared to clopidogrel. No significant difference in bleeding risk was observed in all subgroup analyses comparing prasugrel/ticagrelor and clopidogrel. Comparable results were also obtained using PP approach and propensity score weighting, with or without trimming (Table S4).

## 4 DISCUSSION

The study demonstrated the long‐term clinical benefit of prasugrel/ticagrelor over clopidogrel as part of DAPT in Hong Kong ACS patients using real‐world territory‐wide clinical database. This study showed MACE reduction, and in particular MI reduction, from 1 year to 5 years after index ACS event using prasugrel/ticagrelor compared to clopidogrel. Clinical benefit in MACE or MI reduction was not observed during the first year after index ACS event. The reduction in overall CV death and stroke were also observed.

Various studies reported clinical outcome of ticagrelor/prasugrel compared to clopidogrel in ACS patients using real‐world registry. Despite clinical efficacy of prasugrel/ticagrelor over clopidogrel demonstrated in landmark trials, several observational studies failed to replicate the results. Taking ticagrelor as example, the landmark trial PLATO demonstrated that ticagrelor, compared to clopidogrel, reduced MACE (HR 0.84, 95% CI 0.77–0.92), MI (HR 0.84, 95% CI 0.75–0.95), vascular death (HR 0.79, 95% CI 0.69–0.91) and all‐cause death (HR 0.78, 95% CI 0.69–0.89) at 1 year.^6^ Coons et al. reported in a study of 8127 post‐PCI subjects that ticagrelor did not show reduced MACE compared to clopidogrel (HR 1.05, 95% CI 0.52–2.13) at 1 year.^11^ A comparative observational study in Greece using Greek antiplatelet (GRAPE) registry showed only reduction of all‐cause death using prasugrel/ticagrelor compared to clopidogrel at 1 year (HR 0.61, 95% CI 0.38–0.98), but not MI (HR 0.65, 95% CI 0.27–1.57) or stroke (HR 1.03, 95% CI 0.53–2.00).^12^ Study from Italy using the START antiplatelet Italian registry also revealed that no clinical benefit was observed in CV mortality (aHR 1.07, 95% CI 0.44–2.61) or all‐cause mortality (aHR 1.36 (95% CI 0.69–2.71), using ticagrelor over clopidogrel as DAPT in post‐ACS patients at 1 year.^13^ Focusing in Asian population, a retrospective study in Taiwan showed insignificant results in CV death (HR 0.57, 95% CI 0.26–1.27), MI (HR 1.02, 95% CI 0.34–3.05), and all‐cause mortality (HR 0.98, 95% CI 0.53–1.79) using ticagrelor over clopidogrel in post‐PCI ACS subjects at 1 year.^14^ Wang et al. reported comparison of ticagrelor versus clopidogrel and showed comparable 1‐year MACE risk (ticagrelor 4.0% vs. clopidogrel 3.1%, p = .246).^15^ The insignificant results from our study during the first year post‐index event also in line with the results reported from the previous observational studies, even with the ones from Chinese population.

It was also worth‐noting the clinical benefit of newer P2Y12 antagonists at long‐term, as stated in PEGASUS‐TIMI 54 trial that, ischemic risk could be presented up to a median of 4.7 years after index MI.^16^ The study using Sweden SWEDEHEART registry showed that ticagrelor reduced all‐cause death at 24 months compared to clopidogrel (aHR 0.83, 95% CI 0.75–0.92), yet marginally insignificant in MI reduction (aHR 0.89, 95% CI 0.78–1.01) and stroke (aHR 0.81, 95% CI 0.65–1.01).^17^ Two observational studies in Korea revealed reduced all‐cause mortality and CV death using ticagrelor compared to clopidogrel at 24 months.^9^, ^18^ Our study examined outcomes beyond 1 year up to 5 years after index ACS event. We demonstrated reduced overall MACE (HR 0.60, 95% CI 0.39–0.91), MI (HR 0.54, 95% CI 0.33–0.91) and all‐cause mortality (HR 0.42, 95% CI 0.26–0.68) with prasugrel/ticagrelor over clopidogrel from 1 year to 5 years. The results remained robust even when we considered sensitivity analysis with PP approach. This implicated that the long‐term effect of antiplatelet agents shall be considered, with regards to the thrombotic risk reduction.

In addition, our study showed that there was no increased overall risk of bleeding using prasugrel/ticagrelor compared to clopidogrel in Hong Kong ACS population. The bleeding risk was comparable between prasugrel/ticagrelor and clopidogrel during the first year (HR 1.07, 95% CI 0.60–1.90). Following that, there was reduction of bleeding risk from 1 year to 5 years when newer P2Y12 agents were used (HR 0.46, 95% CI 0.21–1.00). This differed from the reported bleeding risk from previous literatures. While various studies from different localities, including Caucasian and Asian populations, showed insignificant difference in bleeding risk between prasugrel/ticagrelor and clopidogrel, several national‐wide registry observational studies revealed increased risk of bleeding. ^11^, ^13^, ^14^, ^19^ From the GRAPE registry, use of prasugrel/ticagrelor, compared to clopidogrel, increased risk of all BARC bleeding at 1 year (HR 1.70, 1.47–1.97).^12^ In Chinese population, increased risk of bleeding was observed in using ticagrelor versus clopidogrel at 1 year (2.3% vs. 1.0%, p = .015).^15^ Increased bleeding risk of 20% beyond 1 year post‐index event comparing ticagrelor versus clopidogrel was observed from SWEDEHEART registry in Sweden and a nationwide population‐based study in Korea.^9^, ^17^

Gastro‐intestinal tract was reported to be common site of bleeding in previous literatures.^9^, ^14^ Use of PPI was shown to reduce risk of bleeding while not affecting clinical efficacy of P2Y12 agents.^20^ As stated in the clinical guidelines, PPI was recommended in combination of DAPT, especially in patients with high bleeding risk.^1^, ^2^, ^21^, ^22^ Yet only few studies included use of gastric protectants (including PPI or H2RA) in the baseline adjustment. We considered the use of PPI or H2RA at the start of study, while duration of gastric protectant use was also considered and adjusted in the subsequent Cox model, allowing a thorough consideration on the effect of gastric protectant on bleeding risk.

When we compared the reported bleeding rate as calculated from Kaplan–Meier analysis, large difference was observed in the bleeding rate. In a Korean‐based population‐wide study, it was reported that event rate at 2 years of any bleeding was 18.1% with ticagrelor and 15.1% with clopidogrel, while event rate at 2 years of major bleeding was 3.1% with ticagrelor and 2.5% with clopidogrel.^9^ In our study, the reported event rate of bleeding at 2 years was 2.8% with prasugrel/ticagrelor and 4.0% with clopidogrel. The discrepancy may be caused by the definition of bleeding event. While we defined bleeding event as “any bleeding leading to hospitalization”, Yun et al. defined major bleeding as “bleeding necessitating hospitalization” in the aforementioned study.^9^ Despite the difference in the definition of bleeding event, the risk of major bleeding of ticagrelor compared to clopidogrel was still shown to be higher at 2 years in the Korean study (HR 1.18, 95% CI 0.98–1.43).^9^ The bleeding rate of clopidogrel in our study (4.0%) seemed to be higher than in the reported bleeding rate in Korean study (2.5%), leading to opposite direction of hazard ratio in bleeding risk.^9^ The underlying cause leading to different bleeding risk in using clopidogrel despite among same ethnicity of East Asian requires further investigation.

We examined the robustness of results by running the analysis with PP approach. With the PP approach, the addition effect of P2Y12 receptor antagonist switching was considered, with the censorship applied. With the PP approach, the results were similar to the ITT approach. Yet we shall not conclude that the effect of P2Y12 receptor antagonist switching being limited, as there was only limited change with the study cohort when P2Y12 receptor antagonist switching was considered. The reality was that most of the P2Y12 receptor antagonist switching would be initiated due to thrombotic or bleeding event, of which we would have considered “event outcome” in the subject. Therefore, our study was not powered to conclude effect of P2Y12 receptor antagonist switching in the DAPT‐treated ACS patients.

There are several limitations in the current study. Firstly, concurrent medications other than oral anticoagulant, PPI and H2RA were not considered in the analysis. In particular, high intensity statin therapy was recommended in all patients with ACS episode from clinical guidelines to reduce MACE rate.^23^, ^24^ Despite the lack of information in the current study, it was previous shown by Wang et al. that prescribing adherence of statin in Hong Kong ACS post‐PCI patients was up to 90%.^25^ Moreover, in patients receiving PCI with stent insertion, details of PCI including number of stents, type of stent, length of stent and location of stent insertion were unknown with the CDARS data collection. These variables would affect the clinical outcome of patients in addition to the choice of P2Y12 receptor antagonist. As the study was retrospective observational in nature, the medication adherence of patients cannot be assessed. Selection bias would be an issue in retrospective observational study. Despite use of propensity score matching, there could be chance of residual confounding factors.

## 5 CONCLUSION

Compared to clopidogrel, prasugrel/ticagrelor was shown to reduce MACE and risk of MI from 1 year to 5 years after index ACS event in Hong Kong ACS patients. In addition, prasugrel/ticagrelor reduced risk of overall CV death, overall risk of ischemic stroke, overall mortality and mortality from 1 year to 5 years after index ACS event. Reduced bleeding risk with prasugrel/ticagrelor compared to clopidogrel was observed from 1 year to 5 years after index ACS event. To conclude, newer P2Y12 receptor antagonists (prasugrel/ticagrelor) provided better clinical benefit compared to clopidogrel as part of DAPT in Hong Kong ACS patients.

## CONFLICTS OF INTEREST

The authors declare no potential conflict of interest.

## AUTHOR CONTRIBUTIONS

Vivian W.Y. Lee and Amy S.M. Lam designed the study. Vivian W.Y. Lee and Bryan P.Y. Yan assisted in data acquisition logistics. Amy S.M. Lam collected and analyzed the data. Amy S.M. Lam drafted the paper. Vivian W.Y. Lee and Bryan P.Y. Yan reviewed and commented the article.

## Data Availability

The data that support the findings of this study are available from the corresponding author upon reasonable request.
